# Dyssynergic Defecation: A Comprehensive Review on Diagnosis and Management

**DOI:** 10.5152/tjg.2023.22148

**Published:** 2023-03-01

**Authors:** Anahita Sadeghi, Elham Akbarpour, Fatemeh Majidirad, Serhat Bor, Mojgan Forootan, Mohammad-Reza Hadian, Peyman Adibi

**Affiliations:** 1 Digestive Disease Research Institute (DDRI), Tehran University of Medical Sciences, Tehran, Iran; 2 Physical Therapy Department, Tehran University of Medical Sciences Faculty of Rehabilitation Sciences, Tehran, Iran; 3 Department of Gastroenterology, Ege University Faculty of Medicine, İzmir, Turkey; 4 Department of Gastroenterology, Gastrointestinal and Liver Diseases Research Center (RCGLD), Shahid Beheshti University of Medical Sciences, Tehran, Iran; 5 Department of Physical Therapy, Brain & Spinal Cord Injury Research Center, Imam Khomeini Hospital Complex, Tehran University of Medical Sciences (TUMS) Faculty of Rehabilitation Sciences, Tehran, Iran; 6 Gastroenterology and Hepatology Research Center, Isfahan University of Medical Sciences, Isfahan, Iran

**Keywords:** Dyssynergic defecation, constipation, anorectal manometry, biofeedback

## Abstract

About one-third of chronically constipated patients have an evacuation disorder, and dyssynergic defecation is a common cause of the pelvic evacuation disorder. In dyssynergic defecation, the coordination between abdominal and pelvic floor muscles during defecation is disrupted and patients cannot produce a normal bowel movement. The etiology of dyssynergic defecation is still unknown. Although a detailed history taking and a careful examination including digital rectal examination could be useful, other modalities such as anorectal manometry, balloon expulsion test and magnetic resonance or conventional defecography are necessary for the diagnosis. Biofeedback therapy is one of the most effective and safe treatments. Here, we provide an overview of dyssynergic defecation as well as how to diagnose and manage this condition.

**Main Points**

- This study is a comprehensive review of one of the most common and challenging functional disorders of the gastrointestinal tract, dyssynergic defecation (DD).
- This study has a particular focus on the diagnostic approaches.
- It is a brief glance of management methods.
- It proposes a practical clinical pathway and designs an algorithm for the identification and management of patients with DD.

## INTRODUCTION

Chronic constipation is one of the most common gastrointestinal complaints of patients in primary care and gastroenterology practice.^1,2^ The precise definition of constipation depends on the perception of both “patients” and “physicians” of chronic constipation (self-reported symptom-based vs. frequency-based constipation), as normal stool frequency and consistency varies among individuals.^2-4^ While physicians regard constipation objectively with infrequent bowel movements less than 3 times a week,^5^ patients have a broader definition mostly related to the ease of stool passage and consistency.^6^ This discrepancy has challenged the estimation of the actual prevalence.^4,5,7^ Thus, in order to standardize this definition, an international working committee proposed more comprehensive diagnostic criteria for functional defecation disorders.^8,9^ Constipation could be divided into 2 subtypes—functional and structural. Functional constipation includes slow transit constipation, evacuatory disorder Irritable Bowel Syndrome with Constipation (IBS-C).^10-12^ Dyssynergic defecation (DD) is a type of defecatory disorder (Table 1).^13^ In 1985, Preston and Lennard-Jones^14^ first ascribed the symptoms of some constipated patients to the failure of pelvic floor muscles relaxation, which resulted in the sustained contraction of the external anal sphincter on attempted defecation and called it “Anismus,” meaning the spasm of the anus. Since then, many terms have been substituted synonymously for this entity, namely, anal sphincter dyssynergia, pelvic floor dyssynergia, paradoxical pelvic floor contraction, spastic pelvic floor syndrome, paradoxical puborectalis contraction, dyskinetic or non-relaxing puborectalis muscle syndrome, obstructive defecation, and pelvic outlet obstruction.^15-19^ However, some of these terms may seem inappropriate, e.g. the “anismus” mostly implies psychogenic aspects of a disease,^20^ or the phrase “pelvic floor” incorporates the functions of micturition and sexual activity in addition to defecation, which are often intact in this disorder.^11,21^ Finally, in 2006, this condition was named “dyssynergic defecation.”^11^ Dyssynergic defecation (etymology: “dys” = abnormal and “synergia” = coordination) refers to any disturbance of the neuromuscular coordination between abdominal, rectoanal, and pelvic floor muscles, leading to inadequate rectal propulsive forces and/or increased resistance to defecation.^21^

Since DD has different definitions and high prevalence in the community, proper and accurate diagnosis, clinical decision-making, and management of this disorder appear to be important. Therefore, this article provides a comprehensive review of the definition and diagnostic approaches of DD as well as a brief glance of management methods.

### Epidemiology

The estimated prevalence of DD directly depends on the estimated prevalence of chronic constipation, which itself is quite heterogeneous in the literature as only about 1:4 to 1:3 of symptomatic patients seek healthcare.^22,23^ Moreover, the DD diagnosis is only verified by conventional laboratory testing and this may result in false-­positive rates because these tests are not exclusive and also an overlap may be present between different causes.^24^

The prevalence of chronic constipation and DD is quite similar across most geographical regions.^25^ The pooled global prevalence of chronic idiopathic constipation has been estimated at 14% (95% CI: 12-17%) and it was cut in half when restricted to the Rome IV diagnostic criteria.^26^ About one-third of chronically constipated patients have an evacuation disorder, and DD is the most common cause of the evacuation disorder, with a prevalence of 27% to 59%.^27,28^ In Iran, the pooled prevalence of functional constipation is estimated to be about 11% (95% CI: 9.5-12.4%).^29^ Female gender, being at the upper end of the age spectrum, particularly after 65 years of age, lower level of education, and socioeconomic status are considered as risk factors for constipation.^21,25,30-33^ Although chronic constipation more frequent in young female, DD and impaired anal sphincter relaxation are more in the patients who were older and male.^34^ Chronic constipation, including DD, imposes a substantial health-care burden on the community’s economy as well as the quality of life.^35,36^

### Etiology and Pathophysiology

The etiology of DD is not fully understood. Yet, DD is thought to be an acquired but subliminal behavioral disorder of defecation rather than an organic or neurogenic disease. This hypothesis is confirmed to some extent since patients can properly learn to relax the pelvic floor muscles when provided with biofeedback training. A prospective survey showed that DD began later during adulthood in nearly two-thirds of patients, less than half of which was attributable to a particular event and the other half had no identifiable precipitating cause.^21^ A multiplicity, mutual interaction, and overlap may exist among various mechanisms in the pathogenesis of dyssynergia. Dyssynergic defecation has been shown to be a consequence of the following abnormalities: pregnancy and childbirth, trauma especially back injury, bad toilet habits (i.e., sitting on the toilet for a long time) and inappropriate learning of defecation during childhood both for behavioral problems or parent–child conflicts, neurogenic disturbances of the brain-gut axis, rectal hyposensitivity, slow transit constipation, anxiety and/or psychological stress, and history of sexual abuse.^16,21,37^

The normal defecation mechanism relies on anatomical integrity as well as synchronized interaction between the associated muscles and the nervous system.^38^ Body position and stool characteristics can influence normal defecation.^39^

Dyssynergic defecation is the consequence of the inability to coordinate the contractions of anorectal muscles, which leads to difficult and ineffective stool evacuation. This is mostly due to a combination of weak rectoabdominal propulsive force with a failure to relax the puborectalis and external anal sphincter muscles or inappropriate contraction of them.^16^ Based on the rectal and anal canal pressure, DD is classified into specific subtypes. In addition, at least one-half of subjects with DD demonstrate evidence of altered rectal wall contractility and/or abnormal sensory perception due to prolonged retention of stool or the brain-gut axis dysregulation.^40,41^

Despite all the conjectures put forward, there still exist several ambiguities. Although preliminary studies were encouraging about the great role of the contracted anal sphincter, later, it was shown that the elimination of this resistance with myectomy or botulinum toxin injections can help only 10% to 30% of patients to improve.^42,43^ Furthermore, about 20% to 30% of healthy subjects may also exhibit paradoxical anal contraction features.^39,44,45^ Another recent milestone was the observations from a large controlled study that inadequate propulsive forces are distinct from DD’s mechanisms.^46^ And finally, however being indicated as a volitional disturbance since the majority of patients with DD learn to relax the pelvic floor muscles appropriately with biofeedback training,^47^ the visceral dysfunctions do not fit into this framework.^48^

### Clinical Features

Patients may present with a variety of signs and symptoms, suggestive of DD^49^ including chronic constipation, affecting the defecation frequency, stool consistency, and amount of force needed for an evacuation, as follows: less than 3 bowel movements per week, lengthy excessive straining, hard or lumpy stools, a feeling of incomplete evacuation, digital facilitation and vaginal splinting of stool passage, perianal heaviness, and obstruction sensation. The occurrence of infrequent defecation (62%), which is an important physicians’ point of view in DD, was shown to be less inclusive than excessive straining (85%), incomplete defecation (75%), and anal digitalization (65%).^21^ Dyssynergic defecation may also be accompanied by some annoying symptoms like anorectal pain, abdominal discomfort, and bloating. It is important to notify that these symptoms alone do not consistently differentiate DD from the other possible diagnoses.^50-52^ Furthermore, talking about defecation-related matters is usually complicated for patients and they may misrepresent their experiences. In this regard, few clinical tools, such as the Wexner constipation questionnaire, the 2-week stool diary, and Bristol stool form scales have been utilized to overcome misconceptions and adequately illustrate the nature of chronic constipation.^8,53-55^

### Diagnostic Approach

We evaluate the patient to exclude alternative diagnosis and confirm the diagnostic criteria.

### General Issues

The first step in making a diagnosis of DD is to exclude any underlying abnormalities. It must always be borne in mind that chronic constipation can arise from inadequate fiber and liquid consumption, immobility, medications, and metabolic, neurological, or structural disorders. These conditions could be readily identified through careful history, physical examinations, and appropriate tests. The presenting complaint should elicit the duration and nature of constipation as well as the presence of other gastrointestinal (e.g., abdominal pain, bloating, and vomiting) or alarm symptoms (e.g., age >50 years with no previous history of colon cancer screening, unintentional weight loss of ≥4.5 kg, rectal bleeding, a family history of colorectal cancer or inflammatory bowel disease, iron deficiency anemia or positive fecal occult blood test, recent onset of constipation, and severe persistent constipation that is unresponsive to treatment).^8,56,57^

#### Clinical Evaluation and Food and Stool Diary:

To reliably describe one’s changes in bowel habits and keep down any misapprehension of self-reported chronic constipation, daily diaries are obtained routinely.^54^ A standard bowel diary investigates the number of bowel movements per day, stool consistency, level of straining, use of digital maneuvers, feelings of incomplete evacuation, and presence of pain and bloating for 1 or 2 weeks. The Bristol stool form scale is a recognized validated defecation diary tool that is used to sort out patterns in bowel habits.^8^ Therewith, a food diary helps physicians to assess fiber and fluid intake and the number, frequency, and also nutrient content of everyday meals.

#### Digital Rectal Examination:

Digital rectal examination (DRE) can reveal signs of many structural and functional conditions, including a stricture, spasm, tenderness, anal fissure, hemorrhoid, mass, blood, or stool.^58^ In the presence of feces during DRE, stool consistency and patient’s awareness of its presence should be noted to evaluate rectal sensitivity. A meticulous DRE, as a simple and reliable bedside screening tool of DD, can primarily raise suspicion of DD and facilitate an appropriate patient selection for further physiologic testing and treatment.^59^ Digital rectal examination is a fairly accurate test relative to anorectal manometry for identifying dyssynergia, with a sensitivity of 75%, a specificity of 87%, and a positive predictive value of 97%.^59^ However, in some cases, it may not be enough solely and require further evaluation.

At first, while the patient is lying in knee-elbow position, the anal resting tone and squeeze function is examined. Thereafter, the patient is asked to bear down and push as if to defecate, and simultaneously, the physician should focus on perceiving a relaxation feeling in the pelvic floor muscles and a descent sensation in the anal canal along with tightening of abdominal muscles.^60^ Patient with DD has an increased resistance to insertion of the finger (high resting anal tone), pulls the finger in during squeeze (negative squeeze anal tone), fails to relax or paradoxically contracts the sphincter complex, and has a reduced perineal descent during the simulated evacuation.^2,59^ Unfortunately, only a few physicians and trainees perform DRE in clinical practice, and it is often a cursory exam based on insufficient knowledge on how to perform a comprehensive evaluation in order to come up with a diagnosis.^61^

#### Laboratory Tests:

There is inadequate evidence to support or reject the utility of routine screening laboratory tests in the evaluation of patients with chronic constipation.^62^ Thus, only selected individuals with alarm features^56,57^ should undergo these tests. Examples of common tests in order to exclude possible secondary causes of constipation include complete blood cell count, serum glucose, creatinine, calcium, and thyroid function tests.^8^

#### Colonoscopy:

Patients without alarm features do not clearly benefit from endoscopic assessments.^8^ On the contrary, in the presence of alarm signs or in patients over 50 years of age with no prior history of colorectal cancer screening, a high diagnostic yield has been achieved with examinations of the colonic mucosa.^63,64^ About 80% of patients with DD have a normal colonoscopy.^65^ The patients with DD require no further colonoscopic follow-up except for those colonoscopy screening indications that already exist.^66^

### Balloon Expulsion Test

The balloon expulsion test (BET) is a simple, office-based clinical test that is indicated as the first-line screening method of defecation physiology in subjects suspected of rectal evacuatory disorders and should be done immediately before or after the protocol of anorectal manometry and rectal sensory testing.^67,68^ The BET is routinely performed by inserting a 4-cm-long balloon attached to a plastic catheter into the rectum of a patient lying in the left lateral position with hips and knees flexed and then inflating it with 50-60 mL of warm water or air.^69^ The balloon can also be replaced by a Foley catheter or silicone-filled stool-like device such as Fecom. After that, the patient is given privacy and asked to expel the device while sitting on a commode. The ability/inability to expel the simulated stool and the time taken are recorded using a stopwatch. Normal accepted time limit for the healthy evacuation is between 1 and 3 minutes, although, almost all expulsions occur in a timely manner within 1 to 2 minutes.^70-72^ However, the BET methodology has not yet been standardized and the normative cut-off points vary owing to the different techniques, body positions, and types of balloons.^62^ A failure can suggest that DD may exist but is not synonymous with DD.^73^ Various studies evaluating the BET in isolation have identified it as a quite specific (80%-90%) but not so sensitive (about 50%) tool for the evaluation of pelvic floor dyssynergia as compared to anorectal manometry and electromyography.^62,71^ In a recent systematic review and meta-analysis, the sensitivity and specificity were estimated at 70% (95% CI: 53-82%) and 81% (95% CI: 75-86%), respectively, with an overall discriminative ability of 0.84 (95% CI: 0.68-0.93), no matter what the subject positioning (seated vs. left lateral decubitus), the maximum allowed expulsion time between 1 and 5 minutes, or the choice of reference test was and BET could be utilized as a point of service clinical test to screen the functional defecatory disorders.^74^ On the other hand, there is the possibility of false negative results as the balloon may not exactly mimic the regular stool or false-positive results as patients may still not feel comfortable outside the confines of their own toileting environment, even though private.^51,62^ Also, the BET is not sufficient to differentiate between underlying mechanisms of disordered defecation or existing overlaps.^51,62^ Accordingly, this test should be performed and interpreted alongside the results of other tests of anorectal function.^67^

### Anorectal Manometry

Anorectal manometry (ARM) is a valuable diagnostic tool that enables a comprehensive assessment of the pressure activity in the rectoanal region, including the anal pressure at rest, during squeezing and straining, the rectal sensations and compliance, and the rectoanal reflexes.^44^ Anorectal manometry usually assists physicians to diagnose DD through the detection of motor abnormalities of producing insufficient propulsive pressure (≤40-45 mmHg) due to impaired rectal contraction and/or incoordinated movements of rectum and sphincters during attempted defecation due to impaired anal relaxation (<20% reduction in anal pressure) or paradoxical anal contraction.^75^ It also helps to screen responses to biofeedback treatment^20^ and accompanying impairments such as sensory dysfunction (defined as an increased rectal compliance and/or rectal hypo/hypersensitivity).^76^ Anorectal manometry can be carried out using various devices and techniques. The conventional ARM (with solid- or water-perfused or aircharged probes) is the first-used technology to this end. During the last decade, more sophisticated catheters and systems with higher resolution provided by 12 circumferential sensors spaced at 1-cm intervals (HR-ARM), and higher definition enabled by 256 circumferential transducers (3D or HD ARM), were introduced.^77,78^ These advancements allow a greater physiologic resolution, a lesser motion artifact, and a better inter-observer agreement in comparison to the conventional ARM,^79,80^ and accordingly, the underlying pathophysiology in DD (abnormalities of the puborectalis muscle and/or anal sphincters) might be distinctly identified with the assistance of newer methods.^81^

**Figure 1.**
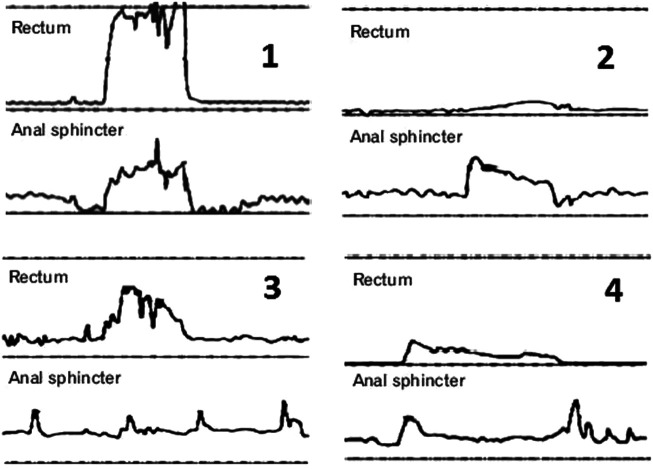
The 4 types of dyssynergic defecation according to anorectal manometry. Type 1 with adequate propulsive pressure more than or equal 40 mmHg and a paradoxical increase anal pressure. Type 2 with inadequate propulsive pressure less than 40 mmHg with a paradoxical increase anal pressure. Type 3 with adequate propulsive pressure more than or equal 40 mmHg and impaired anal relaxation less than or equal 20 mmHg. Type 4 with inadequate propulsive pressure less than 40 mmHg and impaired anal relaxation less than or equal 20 mmHg.

**Figure 2.**
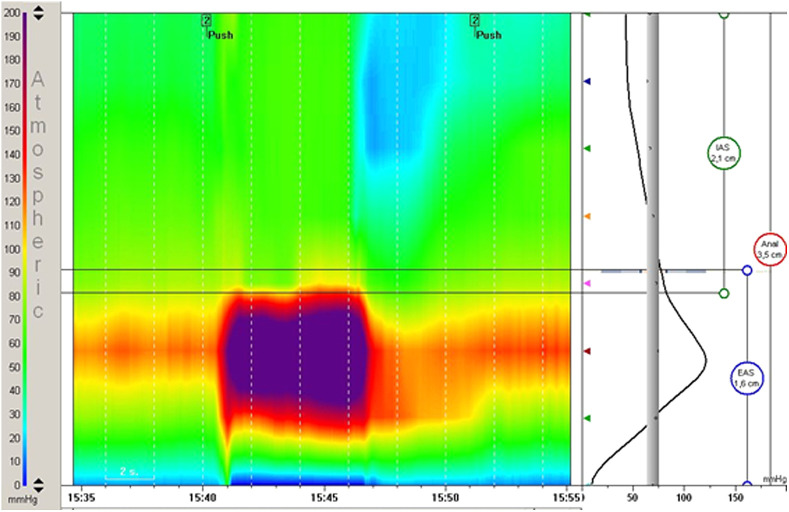
Manometry of a patient with dyssynergic defecation during push period. Intra-rectal pressure did not increased when anal pressure significantly raised (compatible with type 2).

Conventional ARM categorizes patients with defecation disorders described by Rao^20^ into 4 classical patterns (Figure 1), Later in 2016, Rao et al^82^ utilized HR-ARM to further characterize DD by distinguishing the exact location of the involved component of paradoxical outlet contraction, which may be puborectalis muscle alone, external anal sphincter alone, or both together. Figure 2 demonstrates an example of pathologic finding in a dyssynergia case.

Consequently, each of the previous types I and II were proposed to be subdivided into 3 different subtypes, resulting in 8 subtypes of DD, totally. In another recent classification using HR-ARM, Grossi et al^46^ phenotypically categorized patients into 3 groups of high anal pressure, low rectal pressure, and a hybrid of both, which can successfully discriminate normal healthy volunteers from those with constipation. The IAPWG protocol and the London Classification provide standard methods for the description of anorectal disorders.^68,83^ The London classification includes 4 categories: disorder of the rectoanal inhibitory reflex, disorders of anal tone and contractility, disorders of anorectal coordination, and disorders of rectal sensation. The IAPWG classification part 3 or disorders of rectoanal coordination describes DD.^68,83^

In this test, while bearing down, a balloon is inserted to the rectum and records the generated propulsive intraabdominal and rectal pressure, while transducers are applied to the anal sphincter and record the relaxation or inappropriate contraction of the external anal sphincter.^75^ As a means to quantitatively measure the rectoanal coordination during simulated defecation and diagnose DD, few simple and useful metrics have been derived, including the rectoanal gradient, the defecation index, and the percentage of anal relaxation.^16,44,76^ During normal defecation, rectal pressure should be more than anal pressure, then the rectoanal index/ratio should be higher than 1 and the rectoanal gradient should be positive. Intuitively, an index of less than 1 or a negative gradient is considered to have correlation with DD.^51^ The defecation index is a ratio of the maximum intrarectal pressure to the minimum residual anal pressure. A value of less than 1.3 may suggest DD.^84^ The percentage of anal relaxation is calculated as [(1 − residual anal pressure/anal resting pressure) × 100].^85^

Anorectal manometry findings may be affected by the patient’s nonphysiological posture (lying on the left lateral side) or the patient’s feeling of being uncomfortable, which prevents the patient from attaining normal anal relaxation.^86^ As a result, a great number of overlaps (up to 90%) may be seen between patients with DD and those of a healthy population,^46^ and therefore, these findings should be interpreted together with the patient’s symptoms and other diagnostic modalities.^87^

### Defecography and Magnetic Resonance Defecography

Barium defecography is a dynamic radiologic study used to evaluate the anatomic and functional changes of the anorectum and pelvic floor during attempted defecation (Figure 2).^88^ It is indicated as an adjunct modality for identifying potential anatomic causes and functional defecation disorders when both ARM and BET are equivocal, or when patients have normal ARM with prolonged BET times.^12^ Defecography begins by placing approximately 150 mL of semi-solid thickened barium paste, which imitates a soft stool, into the patient’s rectum. The patient is then instructed to sit on a commode (natural defecation posture) adjacent to a video-fluoroscopic imaging system and squeeze or evacuate the barium while simultaneously this process is being recorded on a videotape. It can provide useful information for the diagnosis of structural abnormalities like rectocele, enterocele, sigmoidocele, rectal prolapse, megarectum, and intussusception. Also, it is applied for the diagnosis of functional defecation disorders by dynamic assessment of the anorectal angle at rest and during expulsion or squeezing, the perineal descent during straining or squeezing, the percentage of rectal emptying with defecation, and the anal canal length and diameter. DD is diagnosed by an anorectal angle change of less than 15 to 20 degrees or a paradoxical contraction of the puborectalis or external anal sphincter muscles during defecation, together with an insufficient pelvic floor descent of less than 1 cm, resulting in prolonged retention or inability to expel the contrast material. However, as being an operator-dependent method with methodological differences and poor inter-observer agreement, its overall usefulness has been limited and cannot be relied upon solely.^28,89^

Dynamic magnetic resonance defecography can simultaneously assess global pelvic floor anatomy and external sphincter morphology and also investigate dynamic anorectal motions and evacuation in real time.^90^ Commonly, this test is performed in the lying position using a closed magnet system. Against traditional fluoroscopy, this method is more precise and provides reproducible measurements with better resolution of the pelvic floor and anorectum soft tissues including anal sphincters and puborectalis muscle, and without any radiation exposure as well as anatomical structures.^91,92^ Howbeit, with the drawbacks of being more expensive than the standard defecography and being not widely available, there is an uncertain added clinical value compared to x-ray imaging.^93^

### Colon Transit Study

Determining whether a patient with DD also suffers from concomitant slow transit constipation is a fundamental step to achieve a successful treatment since these 2 may coexist in up to two-thirds of patients.^21^ Furthermore, it is believed that the DD itself is responsible for delayed colonic transit and it has to be either excluded by normal ARM or BET or treated before the whole gut study.^12,51^ Colonic transit study, measuring the time it takes for stool to pass through the colon, can aptly illustrate the overall colonic motor function quantitatively and it can be measured by obtaining abdominal radiographs after patients ingest radio-opaque markers,^94,95^ that is single or multiple capsules technique.^96,97^ A wireless motility capsule (WMC)^98^ or by scintigraphy of a radioisotope-labeled meal and detecting its residual radioactivity.^99^ Interpretation of CTT is based on the identification of retained markers in 3 regions, the right and left colon, and the rectosigmoid region.^100^ Up to two-thirds of patients with a defecation disorder also have delayed colonic transit.^21^ The outlet dysfunction could be responsible for delayed colonic transit and a study showed that colonic transit improved after dyssynergia treatment with biofeedback therapy in patients with both dyssynergia and slow colonic transit.^51^ Hence, treatment of DD is recommended as an initial step in patients with chronic constipation and if biofeedback was not successful, further evaluation such as CTT is recommended.

### Further Evaluations

#### Endoanal Ultrasonography:

Endoanal ultrasonography is rarely used for structural assessment of the anal sphincters in patients with obstructed defecation.^101,102^ A meta-analysis estimated an acceptable concurrent positivity for endosonography among those with positive ARM in patients with DD.^28^

#### Anal Electromyography:

Needle electromyography (EMG) is a neurophysiologic test that can sensitively characterize disturbances in the motor and sensory innervation of the anorectal canal and pelvic floor muscles using fibrillation potentials and reveal any myopathic or neurogenic damages^103^ but this test is rarely used in clinical practice.^104^ This test may be considered in conjunction with pressure measurements to selectively distinguish the affected components in DD with more detailed information about the anatomy and physiology of the pelvic floor. What is more, it is widely used to facilitate coordinated sphincter contraction during biofeedback training for DD.^105^ The average EMG activity of the external anal sphincter and puborectalis muscle is recorded by needle electrodes and is used to identify dyssynergia.^106^ A reduction of <20% in anal EMG activity during an evacuation is probably correlated with DD.^71^

### Diagnostic Criteria for Dyssynergic Defecation

A troublesome challenge in diagnosing functional evacuatory disorders is that none of the aforementioned symptoms or abnormal diagnostic tests alone is reliable as a good predictor of the etiology and pathophysiology in patients with chronic constipation.^51,69^ Hence, the utilized diagnostic criteria for DD must interpret the physiological tests in complementary to the clinical features.^63^ Based on Rome IV criteria, DD is a subtype of functional defecation disorders.^26^

Inadequate defecatory propulsion is the other cause of functional defecation disorders besides DD. Since some patients with irritable bowel syndrome may slightly show degrees of pelvic floor dysfunction,^107,108^ DD can be effectively resolved with biofeedback therapy irrespective of coexistent irritable bowel syndrome.^109^ The latest Rome criteria have been justified to include patients with ­constipation-predominant irritable bowel syndrome, as well.

## Accompanying Abnormalities and Complications

In some patients, DD may go beyond a locoregional disorder and be parallel or causally associated with other physical or mental abnormalities. Understanding these conditions is essential for successful treatment and may prevent the added burden and morbidity of these complications. Similar to other causes of chronic constipation, patients with untreated DD can develop overflow fecal incontinence as a result of dysfunction of the pelvic floor nerves and muscles and retention of impacted feces.^110^ It must be pointed out that the treatment of DD alone may be not sufficient when encopresis has happened, and both should be taken care of.^111^ Besides, prolonged constipation may be complicated by the incidence of megarectum, rectocele, stercoral ulceration, solitary rectal ulcer syndrome, anal fissures, diverticulosis, and hemorrhoids, which can also lead to secondary voluntary stool retention, and thereby, a vicious cycle.^16,112^ In a cohort study endoscopically evaluating patients with DD, it was demonstrated that although most patients revealed normal colonoscopy (81.2%), hemorrhoids, anal fissure, and solitary rectal ulcer syndrome were present in 11.4%, 3.1%, and 2% of them, respectively.^65^ What is more, it is expected that a DD pattern accompanies up to 80% of patients with solitary rectal ulcer syndrome.^113^ Some patients with DD may exhibit features of pelvic floor laxity causing cystocele, excessive perineal descent, and rectal prolapse.^16,92^ Other functional gastrointestinal disorders like gastroesophageal reflux disease and functional dyspepsia,^114^, as well as delayed gastric emptying are anticipated in DD.^114^ However, they all need to be further evaluated. Beyond everything said before, a substantial number of patients with slow transit constipation, irritable bowel syndrome, and levator ani syndrome have important overlapping features of DD, which can be effectively treated with biofeedback therapy.^21,109,116^

Patients with DD also show a significant degree of impairment in mental and social health-related quality of life, as well as general well-being,^21^ which is comparable with that for some chronic organic conditions of life.^36^ On the other hand, these scores improve after the relief of symptoms.^117,118^ Based on limited information available regarding the role of psychosocial factors, patients with DD also have a higher prevalence of several psychological issues such as anxiety, depression, obsessive compulsive disorder, phobia for relapse of painful defecation, somatization, and a history of physical or sexual abuse.^21,119,120^ Psychological distress has demonstrated a negative impact on the outcome of biofeedback therapy.^121^ Since some of these features improve after successful biofeedback treatment, they could be considered as consequences of obstructed defecation rather than a simple concomitant coincidence.^10^

## Proposed Clinical Pathway

**Figure 3.**
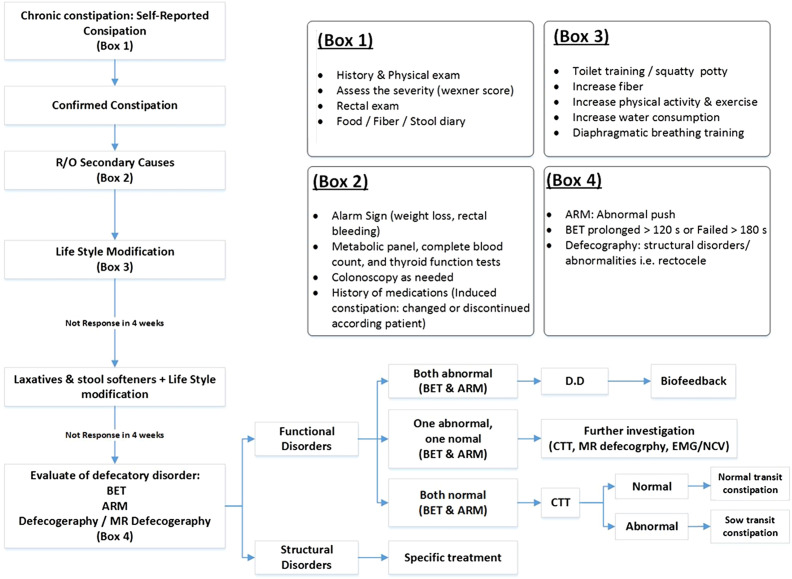
Proposed clinical pathway. R/O, rule out; BET, balloon expulsion test; ARM, anorectal manometry; MR Defecography, magnetic resonance defecography; DD, dyssynergic defecation; CTT, colon transit time; EMG/NCV, electromyography and nerve conduction study.

Based on our experience and the literature, we have proposed a clinical pathway for the identification and management of patients with DD, which is briefly illustrated in Figure 3.

## Management

The DD management is comprised of the standard constipation treatment, biofeedback, and other invasive therapies, such as botulinum toxin injection and anorectal myectomy, and should be individualized based on patients’ symptoms, age, and co-morbid conditions.

### Standard Constipation Treatment

#### Avoiding Constipating Medications:

Following medications may induce constipation and need to be avoided in patients with chronic constipation: aluminum-containing antacids, iron or calcium supplements, non-steroidal anti-inflammatory drugs (NSAIDs, i.e., ibuprofen), antidepressants (i.e., tricyclic antidepressants, TCAs), antipsychotics (i.e., clozapine), antihistamines (i.e., cetirizine), antiepileptics (i.e., carbamazepine), antispasmodics (i.e., hyoscine).^120^

#### Adequate Fluid Intake:

It has been shown that daily consumption of 2 liters of mineral water in patients with chronic constipation, compared to 1, increases the defecation frequency per day.^123^ However, this finding may be confounded by the presence of Magnesium in the water, which has a laxative effect in the water. Currently increased water intake is suggested only in the presence of dehydration.

#### Adequate Fiber Intake:

A high-fiber diet (either natural or supplemental) in patients with chronic constipation may be beneficial by bulking up the stool, absorbing water, and maintaining the healthy gut microbiota composition.^124,125^ Daily consumption of about 25 g of fiber improves symptoms, increases the frequency of defecation, and also reduces laxative use. Disadvantageously, a majority of patients experience bloating especially with a rapid increase in fiber intake, and DD also responds poorly to fiber supplements. Adequate water intake along with fiber supplements would be recommended to avoid hard and bulky stools.^13,63,126,127^

#### Regular Exercise and Physical Activity:

Regular exercise and physical activity may be an effective and feasible strategy in patients with chronic constipation.^128,129^ However, a recent guideline showed that the role of exercise on constipation is uncertain.^130^

#### Timed Toilet Training:

Patients need to be educated to attempt to defecate at least twice a day, after waking up and about 30 minutes after their meal, and to avoid postponing it, when necessary. During defecation, patients should exert 50%-70% of their maximum straining effort for at least 5 minutes.^13,131^

### Pharmacologic Therapy

Four classes of laxatives are commonly used in the treatment of constipation: (a) bulk laxatives such as psyllium, (b) osmotic laxatives such as lactulose, polyethylene glycol, and magnesium hydroxide, (c) stimulant laxatives such as bisacodyl, and herbal laxatives such as senna, serotonin agonists (tegaserod, prucalopride, and velusetrag), secretagogues (lubiprostone, linaclotide, and plecanatide), bile acid-modifying agents (chenodeoxycholate and elobixibat).^132-137^ Almost all the medications listed above have been shown to be superior to placebo in patients with chronic constipation.^138^ Although laxatives and newer agents, namely intestinal secretagogues and serotonergic enterokinetic agents, may not be promising in patients with DD, they can be efficacious if used in combination with biofeedback therapy.^105,139,140^ Table 2 summarized routinely used medications from all of the above mentioned classes.

### Biofeedback Therapy

#### Definition and Goal:

In biofeedback therapy (BFT), data from physiological processes like muscle contraction/relaxation are converted into visual or auditory signals, and the patient can correct his/her function by conditioning techniques.^141,142^ The goal of BFT in patients with DD is to restore the normal pattern of defecation, which is achieved by (a) coordination of abdominal activity with rectal, puborectalis, and anal sphincter muscles by increasing the abdominal pressure while relaxing the pelvic floor muscles and anal sphincters, (b) simulated defecation, and (c) sensory training of the rectum.^143,144^

#### Types of Devices:

Manometric (pressure measurement with solid-state or water-perfused probe) systems, electromyographic (EMG) systems, and home-training devices are used for BFT. Electromyographic probes are cheaper, more durable, and usually give single or dual-channel displays, while more expensive manometric systems provide multichannel displays. Recent randomized clinical trials have shown that home-based device is as effective as office-based devices in improving function and symptoms of anorectal.^145^

#### Treatment Protocol:

First, patients should be explained about the anatomy of the pelvic floor muscles, the physiology of defecation, the importance of diaphragmatic breathing technique, and the correct posture during defecation.^12,145,146^ For the diaphragmatic breathing training, the patient is asked to lie down in a supine position with flexed knee and place one hand on the chest and the other on the abdomen (on rectus abdominis muscle), and do not move the upper chest during breathing. So, the emphasis is on moving the abdomen outward during inhalation and moving it back inward while exhaling.^147,148^ In BFT, the patient is instructed to increase intraabdominal pressure, while decreasing the pressure of the anal canal and prevent paradoxical contraction of the pelvic floor muscles and anal sphincter. Finally, the patient is trained in simulated defecation process using an inflated balloon.^12^ Also, if necessary, sensory training of the rectum should be done.^149^

#### Duration and Frequencies of Treatment Protocol:

In the literature, the number of required sessions, as well as the duration of BFT are different. On average, between 4 to 6 sessions, once or twice a week, that each session lasts between 30 and 60 minutes are recommended for BFT. Also, patients should perform squeezing and relaxing exercises accompanied by diaphragmatic breathing at home 2-3 times per day and each time for 15 to 20 minutes.^142,145,146,150^ In home-based biofeedback protocol, in the first session, the patients are instructed and trained to apply their device at home, so that they can perform bearing down maneuver and anal relaxation at least twice per day and each time for 15 minutes.^144^

#### Efficacy and Outcome Measures:

Studies have clearly shown that BFT is more effective than dietary modification, laxatives, diazepam, muscle relaxants, placebo, and sham biofeedback,^12,150-152^ has longer persistency and no adverse effect.^143^ The efficacy of this method is, of course, too different in children, since it requires a high level of motivation and attention.^12^ Biofeedback therapy could improve bowel movements, stool consistency, straining, sensation of incomplete evacuation, quality of life, and para-clinical features of DD in ARM and BET.^144,153,154^

## Figures and Tables

**Table 1.** Proposed Diagnostic Criteria for Dyssynergic Defecation^13,a^

Patients must satisfy the diagnostic criteria for functional constipation and/or constipation-predominant IBS.	Patients must demonstrate dyssynergic pattern during repeated attempts to defecate.	A dyssynergic pattern of defecation (types I-IV) is defined as a paradoxical increase in anal sphincter pressure (anal contraction), or less than 20% relaxation of the resting anal sphincter pressure, or inadequate propulsive forces observed with manometry, imaging or electromyographic recordings	Patients must satisfy one or more of the following criteria*	Inability to expel an artificial stool (50 mL water-filled balloon) within 1-2 minutes. Inability to evacuate or ≥ 50% retention of barium during defecography.

*Some laboratories use a prolonged colonic transit time, i.e. greater than 5 markers (≥ 20% marker retention) on a plain abdominal radiography taken 120 hours after ingestion of one radio-opaque marker capsule containing 24 radio-opaque markers

^a^Reproduced from *J Neurogastroenterol Motil* 2016;22(3):423 with permission.

**Table 2:** Summary of Routine Medications Recommended According to Available Evidences by Scientific Societies

	American College of Gastroenterology		World Gastroenterology Organization	
	Grade of evidence	Level of recommendation	Grade of evidence	Level of recommendation
Lactulose	Low	Strong	B	2
PEG	High	Strong	A	1
Psyllium	Low	Strong	B	2
Bisacodil	Moderate	Strong	B	2
Senna	N/A	N/A	C	3
Prucalpride	Moderate	Strong	A	1
